# High throughput sequencing of T-cell receptor repertoire using dry blood spots

**DOI:** 10.1186/s12967-019-1796-4

**Published:** 2019-02-18

**Authors:** Shang-Gin Wu, Wenjing Pan, Hongna Liu, Miranda L. Byrne-Steele, Brittany Brown, Mollye Depinet, Xiaohong Hou, Jian Han, Song Li

**Affiliations:** 1 Department of Internal Medicine, National Taiwan University Hospital, National Taiwan University, Taipei, 10002 Taiwan; 2 Department of Internal Medicine, National Taiwan University Cancer Center, National Taiwan University, Taipei, 10672 Taiwan; 3 HudsonAlpha Institute for Biotechnology, 601 Genome Way, Huntsville, AL 35806 USA; 4 iRepertoire Inc., 800 Hudson Way Suite 2319, Huntsville, AL 35806 USA; 5 iCubate Inc., Huntsville, AL 35806 USA

**Keywords:** T cell receptor, TCR repertoire, CDR3, Dry blood spots, Next-generation sequence, Diversity

## Abstract

**Background:**

Immunology research, particularly next generation sequencing (NGS) of the immune T-cell receptor β (TCRβ) repertoire, has advanced progression in several fields, including treatment of various cancers and autoimmune diseases. This study aimed to identify the TCR repertoires from dry blood spots (DBS), a method that will help collecting real-world data for biomarker applications.

**Methods:**

Finger-prick blood was collected onto a Whatman filter card. RNA was extracted from DBS of the filter card, and fully automated multiplex PCR was performed to generate a TCRβ chain library for next generation sequencing (NGS) analysis of unique CDR3s (uCDR3).

**Results:**

We demonstrated that the dominant clonotypes from the DBS results recapitulated those found in whole blood. According to the statistical analysis and laboratory confirmation, 40 of 2-mm punch disks from the filter cards were enough to detect the shared top clones and have strong correlation in the uCDR3 discovery with whole blood. uCDR3 discovery was neither affected by storage temperatures (room temperature versus − 20 °C) nor storage durations (1, 14, and 28 days) when compared to whole blood. About 74–90% of top 50 uCDR3 clones of whole blood could also be detected from DBS. A low rate of clonotype sharing, 0.03–1.5%, was found among different individuals.

**Conclusions:**

The DBS-based TCR repertoire profiling method is minimally invasive, provides convenient sampling, and incorporates fully automated library preparation. The system is sensitive to low RNA input, and the results are highly correlated with whole blood uCDR3 discovery allowing study scale-up to better understand the relationship and mutual influences between the immune and diseases.

**Electronic supplementary material:**

The online version of this article (10.1186/s12967-019-1796-4) contains supplementary material, which is available to authorized users.

## Background

Immunology research, particularly next generation sequencing (NGS) of the immune T-cell receptor β (TCRβ) repertoire, has advanced progression in several fields including treatment of various cancers and autoimmune diseases [1]. The advantage of immunotherapy in cancers has led to increasing numbers of studies dedicated to exploring the impact and interaction between immunity and cancer cells. However, the complexity of the immune system in combination with the limitation of detection methods makes this subject difficult to research.

Currently, TCR repertoire sequencing is widely used to evaluate the immune system [2]. TCR repertoires of patients were explored in a variety of disorders—patients under cancer immunotherapy, autoimmune disease [rheumatoid arthritis (RA), ankylosing spondylitis], or subject to virus infection (hepatitis, human immunodeficiency virus (HIV)) [3–7]. Analyzing the TCR repertoire may help to gain a better understanding of the immune system features and of the etiology and progression of diseases, in particular those with unknown antigenic triggers. Rapid progress has been made in the deep profiling of TCR repertoires by using NGS to discover millions of sequences from the TCR repertoires [8]. There are different TCR library preparation for NGS, including: multiplex PCR, targeted enrichment methods, 5′Rapid amplification of cDNA ends (5′RACE) cDNA synthesis, template-switch, and nested PCR [9]. For TCR repertoire assessment, the current standard sample type is total RNA extracted from whole blood by venipuncture. However, the costs, participant burden, regulatory constraints, and logistics associated with venipuncture and RNA handling are major barriers to clinical application or community-based research on various diseases.

Dry blood spots (DBS) have been used broadly in disease screening, drug level monitoring, and infectious microorganism detection, such as the detection of HIV and plasmodium [10, 11]. There are two major advantages of this method. First, it is minimally invasive (requiring only 0.3 mL of capillary blood obtained by finger- or ear-prick) and a field-friendly alternative to venipuncture by professional medical staff. Second, it is low cost and convenient to handle in that the filter cards can be stored and shipped at room temperature without refrigeration for at least 1 month [12, 13].

The desire for TCR repertoire profiling in a variety of diseases and the advantages of a DBS-based method prompted us to establish a DBS-based TCR repertoire profiling method. Here, we report for the first time the use of a DBS sample in combination with a fully automated and closed system for TCRβ chain NGS library preparation. We have demonstrated that storage of DBS in filter cards up to 28 days at either room temperature or − 20 °C has no effect on unique CDR3 (uCDR3) discovery. Compared with whole blood, the DBS-based TCR repertoire profiling method is minimally invasive, provides convenience when sampling, is sensitive, and is highly correlated with whole blood in terms uCDR3 discovery, facilitating ease of incorporation and scale-up to studies which seek to explore the relationship between immunity in variety diseases.

## Materials and methods

### Subjects and sample collection

All enrolled subjects had written informed consent prior to the collection of whole blood or DBS via finger-prick. The study design and recruiting of patients were approved by the New England Independent Review Board^®^ (NEIRB) (IRB Number: 14-378). All experiments were performed according to the relevant guidelines and regulations.

Whatman FTA 903 cards (Sigma-Aldrich Corp. MO, USA) were used for collection of capillary blood obtained by finger-prick. In addition, peripheral blood was collected via venipuncture for comparison. The Whatman cards were stored in zip-lock plastic bags with a desiccant (ULINE. Atlanta, USA), at room temperature or − 20 °C (for longer storage) for future studies.

### RNA extraction from DBS and whole blood

RNA extraction from DBS was performed following the methods proposed by Karlsson et al. [14]. 2-mm disks were punched out from a DBS with a sterile Robbins true-cut disposable 2-mm biopsy punch (Robbins Instruments, Inc., NY, USA). The punch disks were then incubated in 700 μL RLT buffer plus β-mercaptoethanol (β-ME). The tubes were then incubated at 37 °C for 60 min in a thermomixer rotating at 1000 rpm. Following incubation, RNA was extracted with a RNeasy Micro Kit (Qiagen, Valencia, CA, USA) according to the manufacturer’s instruction. For whole blood, a RNeasy Mini Kit (Qiagen, Valencia, CA, USA) was used according to the manufacturer’s protocol. RNA concentrations were measured by spectrophotometry.

### RT-PCR and sequencing of T-cell receptor β-chain CDR3 region

iRepertoire multiplex primer sets (iRepertoire, Inc. Huntsville, AL, USA) were used to amplify the CDR3 region of TCRβ chain by using RNA as template as described by Wang et al. [15]. The whole amplification process and library preparation process for NGS were fully automated in the iR-Procecessor and iR-Cassette (iRepertoire, Inc. Huntsville, AL, USA). Then, paired-end sequencing was performed on purified PCR products using an Illumina MiSeq v2 300-cycle Reagent Kit (Illumina Inc.), for an average read depth of 30,000 reads per sample.

### Sequence data analysis

Raw cDNA sequences were first analyzed to identify V and J genes by using iR-map and visualized in iRweb (iRepertoire, Inc. AL, USA). Analyzed data from iRweb include peptide sequences, alignments to the international ImMunoGeneTics (IMGT) database, uCDR3, shared CDR3s, and V- and J-gene usage. Multiple alignments and hierarchical clustering of conserved amino acid sequences were analyzed as described by Wang et al. [15].

### Computational error correction of bulk TCR sequences by replicates

Errors in sequencing resulting from PCR errors, PCR contamination and read error were mitigated according to the modified method of Glanville et al.’s study [16]. RNA samples were split into two reactions and processed as technical replicates. The coefficient of determination (*R*^2^) was calculated by linear regression to show the correlation between the replicates of TCRβ CDR3 frequencies prior to data analysis to exclude PCR and sequence errors.

### TCR repertoire diversity analysis

The diversity of the TCR repertoire was calculated based on the diversity 50 (D50) value and the Diversity Index (DI) [17]. Briefly, D50 is the percent of dominant T cell clones that account for the cumulative 50% of the total CDR3s counted in the sample. The mathematical formulation of D50 is defined as follows:

$${\text{D}}50 = \left( {{\text{No}}.\;{\text{of uCDR}}3{\text{ that make up }}50\% {\text{ of the total reads}} \times 100} \right)/{\text{No}}.{\text{ of uCDR}}3{\text{s}}$$

The more diverse a library, the closer the value will be to 50.

The DI is defined mathematically as follows:

$${\text{Assume}}\;{\text{that the numbers }}\left( {\text{n}} \right){\text{ of uCDR3}}{:}\;r_{1} \, \ge \,r_{2} \, \ge \,r_{3} \, \ge \, \cdots r_{i} \, \ge \,r_{i + 1} \ge \cdots \, \ge \,r_{n}$$

where r_i_ is the frequency of the i-th CDR3 and n is the total number of unique CDR3s.

$$x_{k} = \frac{k}{n},\;\;\;\;\;y_{k } = \frac{{\mathop {\sum\nolimits_{i = 1}^{k} {r_{i} } }\nolimits_{{}}^{{}} }}{{\sum\nolimits_{i = 1}^{n} {r_{i} } }}$$

In addition, tree maps and two-dimensional (2D) heat maps were used to reveal the diversity and characteristics of TCR repertoire. In a tree map, each rounded rectangle represents a unique entry: V–J combination of uCDR3, where the size of a spot denotes the relative frequency. 2D heat maps showed that the relative frequency of a consensus germline V-gene allele (as per alignment with the IMGT database) is plotted relative to the consensus germline J-gene allele. Therefore, it is immediately evident which V–J combination is used either frequently or infrequently by the color of the map.

### Statistical analysis

Analysis was performed using the Statistical Package for the Social Sciences (SPSS) 17.0 software. Chi-squared test was performed on all categorical variables, with the exception of those with an expected frequency of < 5, which were analyzed by Fisher’s exact test. Two-sided Student’s *t*-tests was used for comparing the means of two independent variables. One-way analysis of variance (ANOVA) was used to analyze differences in the number of uCDR3s among the different storage durations.

## Results

### T-cell repertoire can be detected from the extracted RNA from DBS

**Fig. 1:**
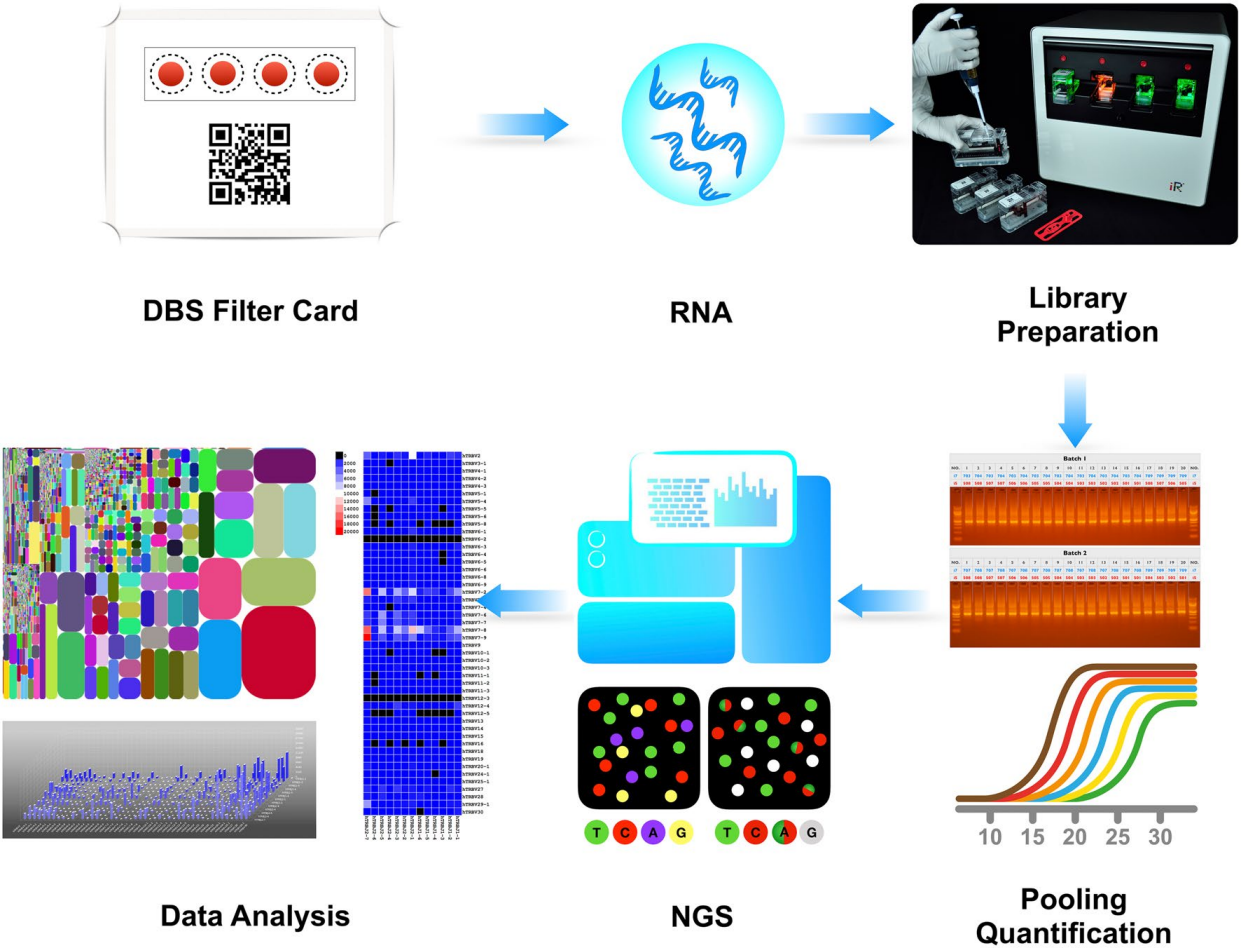
The work flow of DBS based TCR repertoire discovery. Finger-prick blood is collected onto the filter card and extracted RNA from DBS is used as a template for amplification. The entire PCR amplification and library preparation are automated in a closed cassette. Post amplification, libraries are pooled and quantified then sequenced by NGS. Sequencing data is analyzed by iRweb’s bioinformatics platform

The work flow of the DBS-based TCR repertoire discovery is shown in Fig. 1. RNA was extracted from DBS, followed by RT-PCR in a fully automated closed system to amplify the TCRβ library. After cDNA pooling of the TCRβ library and quantification, NGS sequencing was performed, and the data was analyzed as described previously.

Since the percentage of dominant clones detected from DBS is highly dependent on TCR diversity of samples, we first calculated the number of punch disks required for dominant clone detection from whole TCR repertoire. Both confidence levels and intervals were calculated to compare TCR repertoire discovery between methods utilizing DBS versus 10 mL of whole blood. According to the normal range of differential count of complete blood cells, 20–35% of white blood cells are lymphocytes. T cells account for 70–85% of lymphocytes [18]. Therefore, there were about 700–2675 T cells per μL of whole blood for one man with 65 kg of body weight with normal white blood count range from 5000–9000 WBC/μL. According to the Whatman card specifications, one circle with a diameter of 0.5 in. can absorb 75–80 μL of blood; therefore, one 2-mm punch disk contains approximately 2 μL of blood. For individuals whose dominant clones comprise 60% of their total TCR repertoire, 80 μL of blood collected on 40 2-mm punch disks could provide 90% confidence level and 9% confidence interval. The volume of blood required for a given confidence level and interval is presented in Additional file 1: Table S1.

**Fig. 2:**
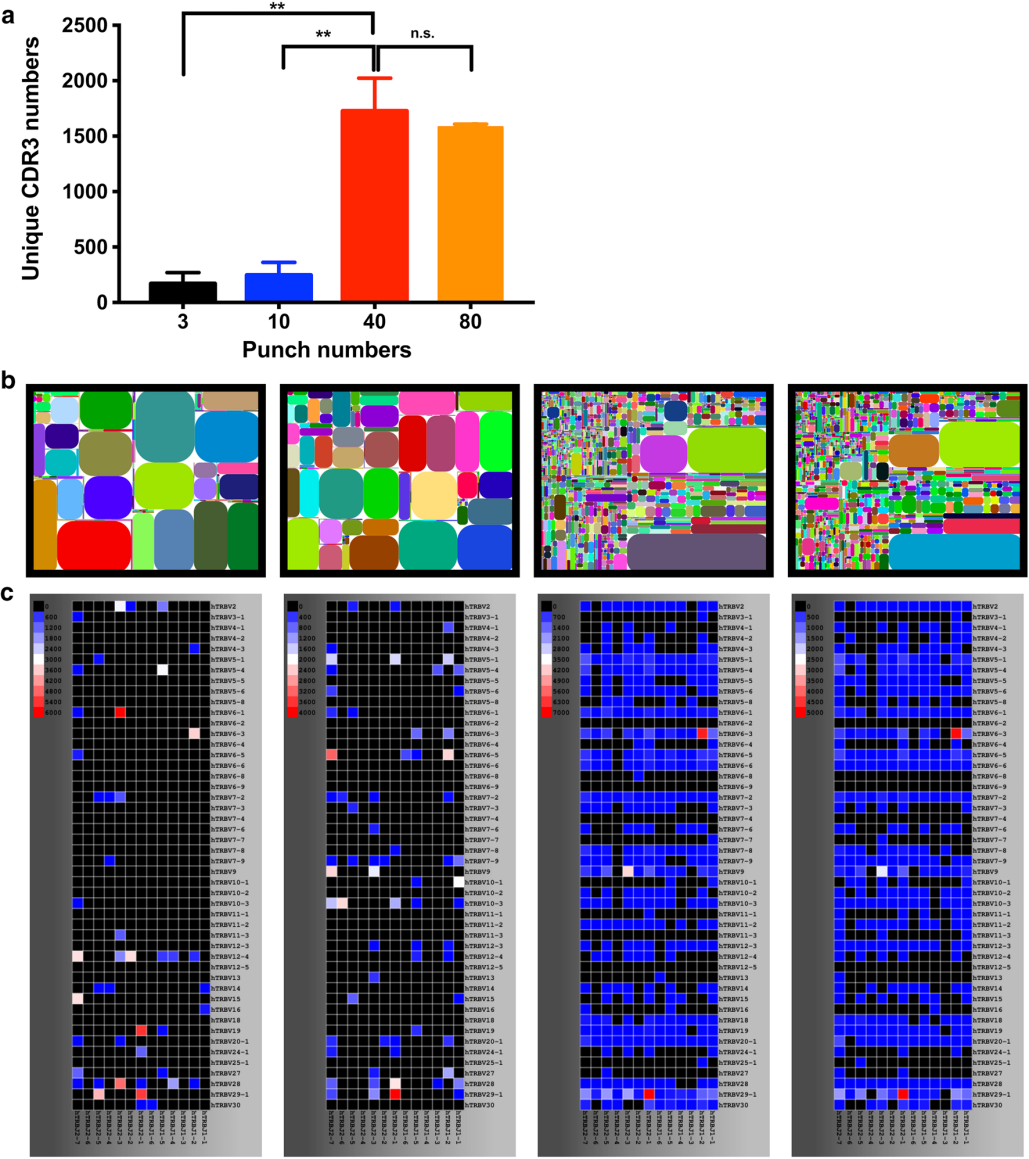
T-cell repertoire is detected from the extracted DBS RNA. **a** uCDR3 discovery from different amounts of punch disks (**p < 0.01, by Student’s t-test). **b** TCR repertoire diversity is illustrated in tree maps where each rounded rectangle represents a unique entry: V–J–uCDR3 and the size of the spot denotes the relative frequency. **c** The relative frequency from low (black) to high (red) of V–J gene combinations, is illustrated in the 2D heat maps from varying amounts of punch disks (left to right: 3, 10, 40 and 80 disks). ns indicates no significant statistical difference

Having calculated the punch disk numbers required for TCR repertoire discovery, we compared between 3, 10, 40 and 80 punch disks. The average uCDR3 discovery for 3, 10, 40, and 80 disks were 169, 246, 1726, and 1581, respectively, with an average read depth of 30,000 reads per sample. The uCDR3 discovery from 40 disks was higher than those from 3 (*p* < 0.0001) or 10 punch disks (*p* < 0.0001). There was no significant statistical difference in average uCDR3 numbers between 40 and 80 punch disks (*p* = 0.520) (Fig. 2a). The tree maps and heat maps also revealed that the diversity and the identified combination of V- and J- gene segments were obviously higher in 40 or 80 punch disks than those in 3 or 10 punch disks (Fig. 2b, c). As a result, 40 disks were established as the optimum punch number for future studies.

### CDR3 frequency correlation between DBS and whole blood

**Fig. 3:**
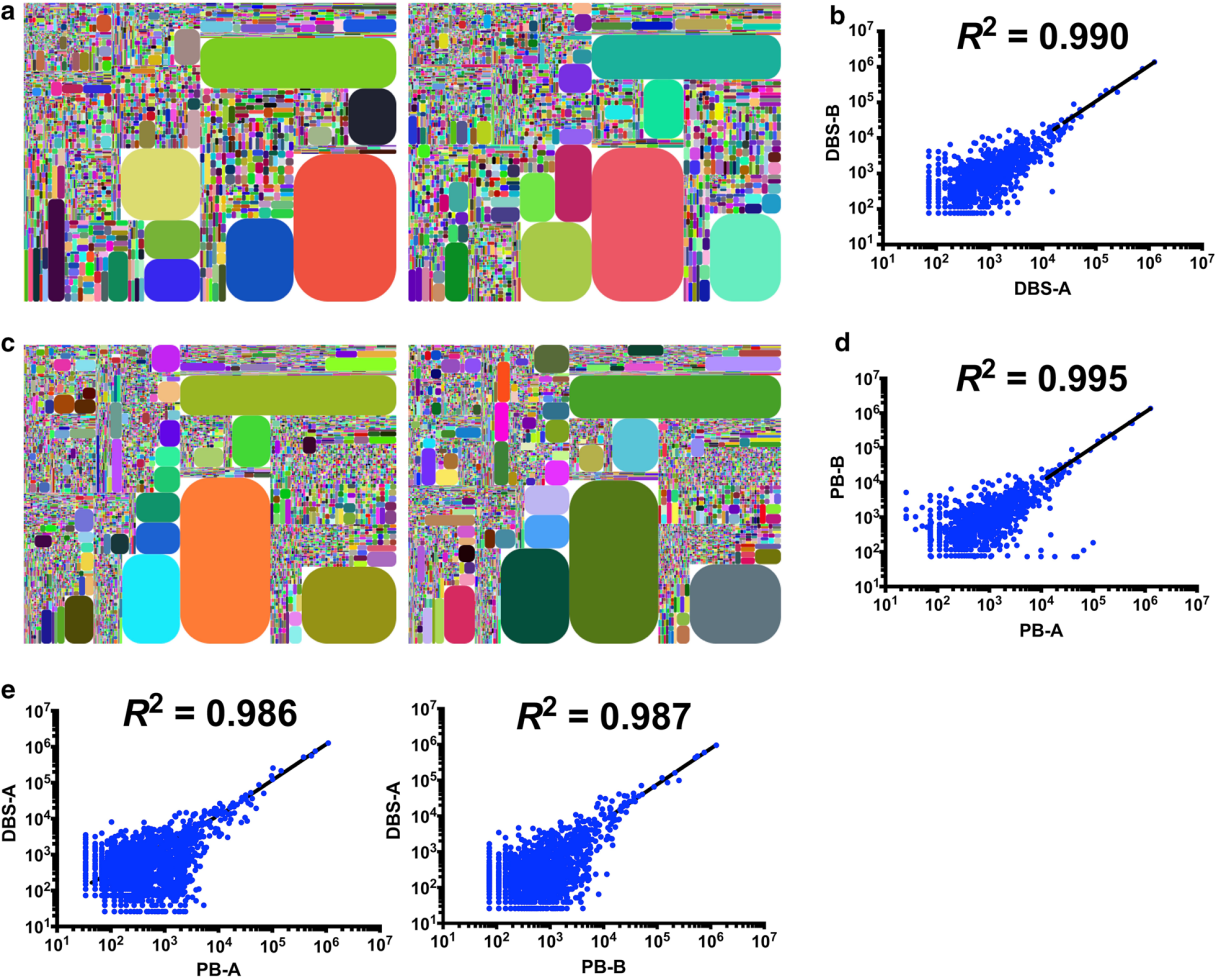
The comparison of TCRβ CDR3 between DBS and whole blood. **a** Tree maps of TCRβ CDR3 from duplicate DBS samples. **b** The coefficient of determination (*R*^2^) of linear regression in TCRβ CDR3 sequences from duplicate DBS samples. **c** Tree maps of TCRβ CDR3 from duplicate peripheral blood samples. **d** The *R*^2^ of linear regression in TCRβ CDR3 sequences from duplicate peripheral blood samples. **e** The *R*^2^ of linear regression in TCRβ CDR3 sequences between peripheral blood and DBS

The *R*^2^ of uCDR3 frequencies were calculated by linear regression between technical replicates to exclude PCR or sequencing error. The *R*^2^ of the regression for CDR3 discovery between technical replicates for DBS and whole blood samples were 0.990 and 0.995, respectively (Fig. 3a–d). uCDR3 frequency correlations were then determined to compare discovery between DBS and whole blood samples. uCDR3 discovery between DBS and whole blood revealed high regression correlation (*R*^2^ = 0.986 and *R*^2^ = 0.987, respectively) (Fig. 3e), with 94% and 92% of the top 50 and top 100 dominant clones from whole blood being detected from DBS, respectively (data not shown).

### Effect of storage temperature and duration on uCDR3 discovery

**Fig. 4:**
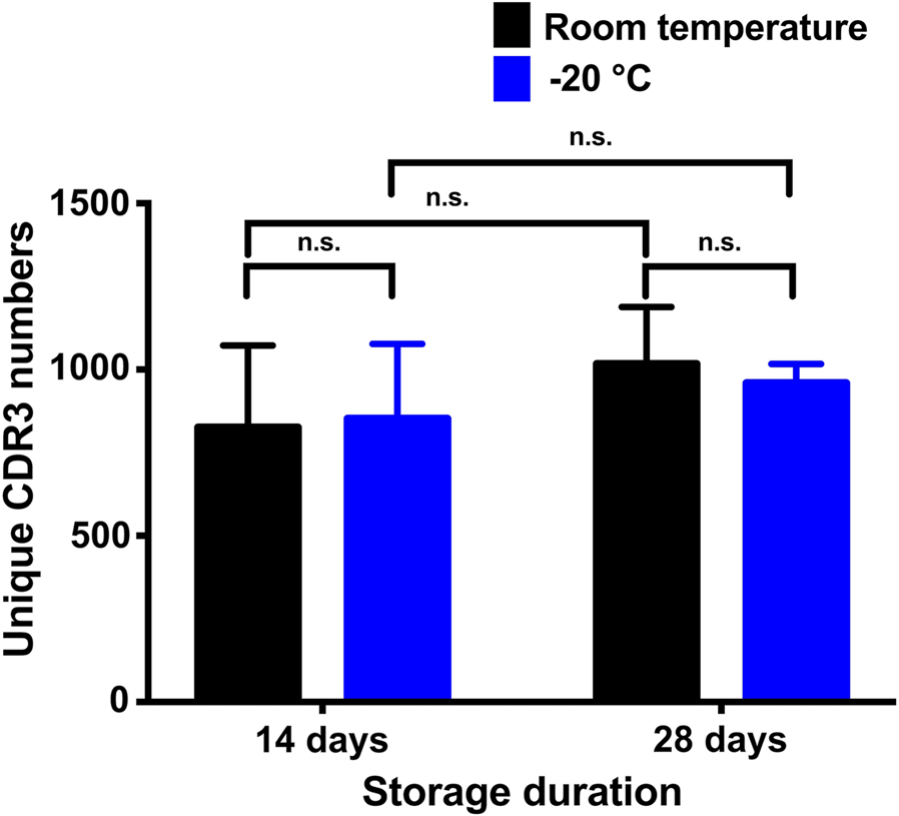
The comparison of uCDR3 discovery between different storage temperatures (room temperature versus − 20 °C). Student’s *t* test was used to determine that there was no significant difference in uCDR3 discovery between the two storage temperatures at both 14 days (*p* = 0.922) and 28 days (*p* = 0.700) storage duration. ns indicates no significant statistic difference

Despite the manufacturer’s suggested storage temperature of − 20 °C, prior studies show conflicting results for the effect of DBS storage temperature and duration on RNA preservation [19–22]. In this study, we compared the uCDR3 discovery between two storage temperatures (room temperature and − 20 °C). No significant difference was demonstrated in uCDR3 discovery between DBS samples stored at room temperature and − 20 °C for 14 days (*p* = 0.922) or 28 days (*p* = 0.700) (Fig. 4).

**Fig. 5:**
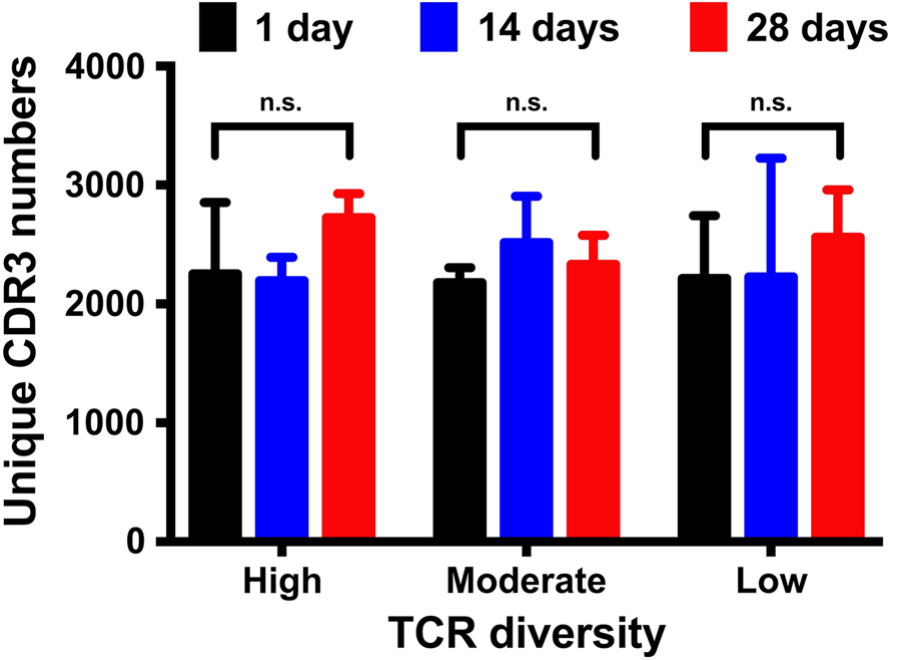
Comparison of uCDR3 discovery with storage durations of 1, 14 and 28 days from DBS samples with varying TCR diversities. There was no significant difference in TCRβ uCDR3 discovery between storage durations for samples with varying TCR diversities by one-way ANOVA analysis. ns indicates no significant statistic difference

uCDR3 discovery from storage duration tests (1 day, 14 days and 28 days) was also compared in DBS samples with low, moderate, and high CDR3 diversities. Among different storage durations, no significant differences were found in uCDR3 discovery in DBS samples with low (*p* = 0.190), moderate (*p* = 0.077), and high (*p* = 0.857) uCDR3 diversities (Fig. 5), and this is true even under the two different storage temperatures tested, room temperature, and − 20 °C (Fig. 4). The uCDR3 frequency did not decrease over time. These results indicate that storage duration of 1, 14 and 28 days had no impact on uCDR3 discovery.

**Fig. 6:**
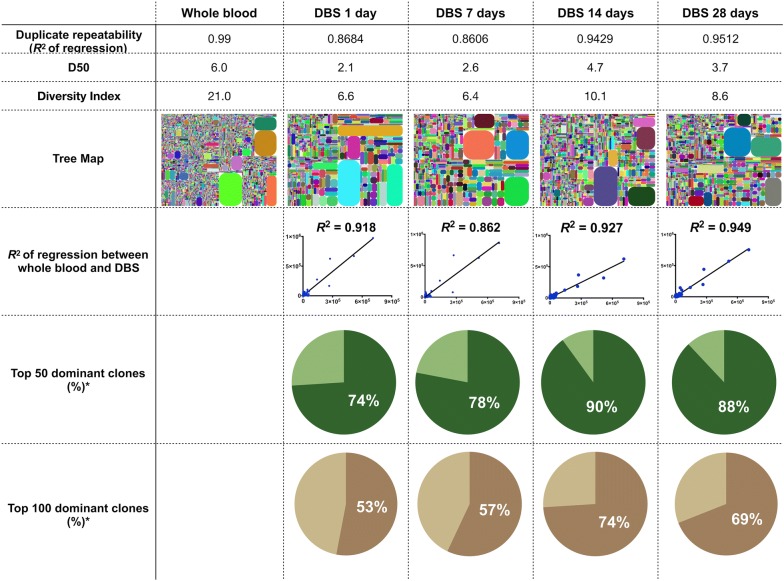
Comparing uCDR3 discovery between different storage durations (1, 7, 14 and 28 days). The *R*^2^ of TCRβ CDR3 sequences was calculated by linear regression between the whole blood and DBS in different storage durations. The *R*^2^ of duplicate sequences showed high correlation in whole blood and DBS within different storage durations. The TCR diversities were shown by D50s, Diversity Indexes and tree maps. There was no significant difference in TCR diversity between storage durations. In addition, more than 74% of the top 50 dominant CDR3 clones of whole blood was detected by DBS. These results indicated that TCR uCDR3s of DBS had high correlation with those of whole blood. (*R*^2^: coefficient of determination calculated by linear regression; *The percentage of TCR dominant clones from whole blood could be detected from the DBS sample)

We also demonstrated the relationship of TCRβ clonotype discovery between DBS and whole blood (Fig. 6). The *R*^2^ of the regression for CDR3 discovery between technical replicates for whole blood samples and DBS in different storage duration tests were 0.990 and 0.86–0.95, respectively. Long storage duration up to 28 days not only showed no impact on uCDR3 discovery, but also showed no impact on the correlation uCDR3 discovery between DBS and whole blood. In addition, the top 50 and 100 dominant CDR3 clones of whole blood could be detected by DBS for 62–88% and 45–69% of the dominant unique CDR3s at 28-day storage, respectively (Fig. 6).

### Relationship between TCR repertoire diversity and uCDR3 discovery

The diversity of each sample’s TCR repertoire can impact the correlation of uCDR3 discovery between DBS and whole blood samples. To explore this relationship, uCDR3 discovery was compared between DBS and whole blood samples known to have low, moderate, and high diversities of TCR repertoire. Table 1 shows the characteristics of TCRβ CDR3 between DBS and whole blood. The samples with low and moderate TCR diversity have a higher regression correlation between DBS and whole blood samples than those with higher TCR diversity (*R*^2^ = 0.989 for low diversity, 0.865 for moderate diversity and 0.806 for high diversify). 92% and 87% of the top 100 uCDR3 clones from whole blood could be detected in a DBS sample of low and moderate diversity, respectively, but only 74% of those in high diversity samples. For samples with high TCR diversity, the top 50 uCDR3 clones from whole blood could still be detected for 82% in a DBS sample. Although the diversity of samples had impact on the correlation of uCDR3 discovery, there was still a relatively high amount of shared uCDR3s between DBS sample and whole blood sample.

**Table 1:** The clinical characteristics of samples with low, moderate and high TCR repertoire diversities

Diversity	Low		Moderate		High	
Sample	WB	DBS	WB	DBS	WB	DBS
Duplicate sequence repeatability (*R*^2^)	0.994	0.991	0.993	0.956	0.930	0.699
Unique CDR3	43,653	5327	38,431	3820	25,118	3208
D50	0.3	1.1	2.7	7.6	14.5	6.8
Diversity Index	15.8	13.2	20.6	16.8	27.1	11.1
*R*^2^ of regression between DBS and WB		0.989		0.865		0.806
Top 50 dominant clones (%)^a^		94%		98%		82%
Top 100 dominant clones (%)^a^		92%		87%		74%

*WB* whole blood, *DBS* dry blood spot, *R*^2^ coefficient of determination calculated by linear regression

^a^The percentage of TCR dominant clones from whole blood could be found from DSB examination

### uCDR3 sharing between samples

In order to determine whether the TCRβ CDR3s detected by this method are unique across individuals, the TCRβ CDR3 sharing was compared among different individuals. The sharing rate of uCDR3 between two individuals was low, ranging from 0.03% to 1.55% (Table 2 and Additional file 1: Table S2). The uCDR3 sharing rate between P5 and P6 is the highest; however, this is a parent–child relationship.

**Table 2:**
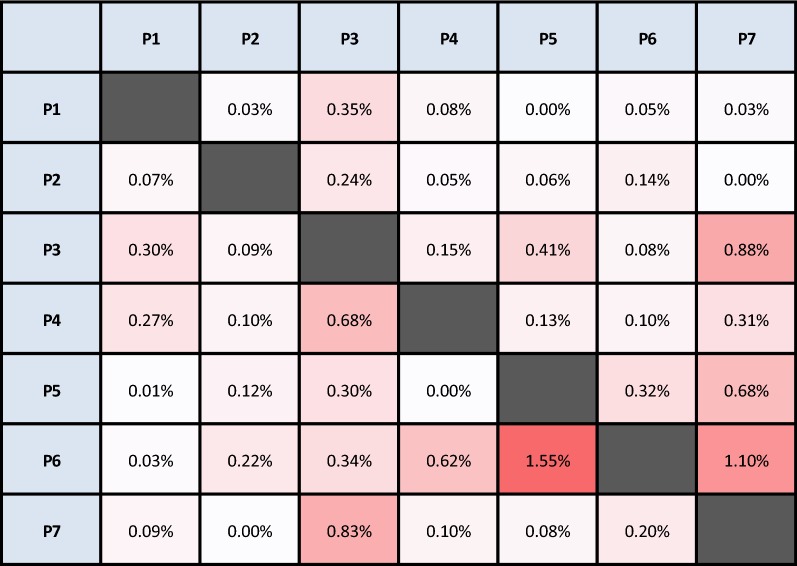
The ratio of the shared uCDR3 reads divided by the total reads of the individual

## Discussion

In this study, we reported for the first time the establishment of a DBS-based method as a minimally invasive alternative to venipuncture blood sampling for TCR repertoire studies. In addition to its cost effectiveness, we have demonstrated that the storage temperature and storage duration had no effect on uCDR3 discovery. The ease of sample collection and the convenience of storage for transportation purposes makes it a viable option for scale-up studies related to TCR repertoires.

The FTA cards entrap nucleic acids and are stabilized at room temperature, which allows long-term storage [20, 23, 24]. DNA extracted from the stored card yields adequate quality and amount for PCR amplification compared with that extracted from the fresh samples [25, 26]. The extracted DNA from the card can also be used for high-throughput molecular analysis [13, 27]. In addition, DBS also provides an alternative tool to formalin-fixed paraffin-embedded cell blocks for biobanking to detect epidermal growth factor receptor (*EGFR*) or Kirsten rat sarcoma 2 viral oncogene homolog (*KRAS*) mutation for personalized target therapy in lung cancer patients [28]. Therefore, this method can be utilized in multiple fields, not only cancer diagnosis but various disease etiologies. In addition to the stability of high-quality nucleic acids, the low cost of storage, and ease in transportation, FTA cards serve as an alternative to various traditional storage methods [20].

We established a method that uses RNA extracted from DBS for high-throughput sequencing. The entire process of RT-PCR amplification and library preparation of the immune repertoire was conducted automatically in cassettes. Automated amplification in a closed, contained environment throughout the entire process prevents contamination and improves the consistency of the results by avoiding error or variation, which may be introduced during manual processing. TCR repertoires derived from DBS are directly comparable with the dominant clones of TCR repertoire discovery from whole blood. As a result, DBS may be used as a screening tool for certain diseases or for genetic screening instead of whole blood.

Peluso et al. used FTA cards to store fine needle aspiration cytology samples of lymph node, and they amplified immunoglobulin heavy and light chains, TCR-β and γ chains by PCR from DNA [29]. The results showed that the amplified DNA from FTA cards is comparable to those of cryopreserved samples. However, a tremendous diversity of TCR repertoires results from extensive recombination, splicing and post-transcriptional processing to yield functional proteins. TCR repertoire discovery from RNA rather than genomic DNA prevents the inclusion of nonproductive or functionally irrelevant sequences [30]. There is currently no study report on the direct detection of the TCRβ repertoire from finger-pricked blood stored on FTA cards.

Although it is known that high-quality DNA can be extracted from the FTA cards [25, 31], there are few studies about RNA extracted from FTA cards compared with other preservation methods. Instability of RNA is a major concern with storage, temperature, and extraction methods. The World Health Organization (WHO) suggests that for HIV drug resistance, dried blood spot specimens should be transferred to − 20 °C or lower as soon as possible although they can be kept and/or transported at ambient temperature up to 14 days after collection [10]. However, Bertagolio et al. demonstrated that a 90% positive amplification rate was noted under the ambient storage temperature of DBS [19]. This is compatible with the current DBS study which shows comparable numbers of uCDR3s between disparate storage temperatures of room temperature and − 20 °C, even after 28 days of storage duration. Therefore, DBS can be stored and transported at room temperature without impacting TCRβ clonotype discovery.

Approximately 74–98% of the top 50 dominant clones from the whole blood are found in the DBS sample. This result indicates that the DBS-based immune repertoire profiling is very sensitive. According to the statistical analysis of sample sizes and the results of the current study, we are now confident that our DBS-based method can reflect whole blood TCRβ repertoire sampling with very good coverage and high sensitivity, i.e., enough shared top clonotypes and strong correlation in uCDR3 discovery.

The recent technological advances in the fields of immunology and immunotherapy hold promise for patients with cancers [32–34]. The TCR repertoire plays a pivotal factor in immunity [35]. For instance, TCR repertoire profiling has the potential to serve as a biomarker of treatment response in pancreatic ductal carcinoma patients who received immunotherapy [7]. Through recognition of major histocompatibility complex (MHC)-peptide complex of TCRs, T cells can be activated and specific T cell clones expanded to give a response to foreign pathogens or cancer cells [36].

Accumulating evidence suggests that immune cells play pivotal roles in a variety diseases and their overlapping regulatory mechanisms in addition to cancers, and TCR repertoire were also explored in different area. For example: Muraro et al. used TCR repertoire to follow the effect of autologous stem cell transplantation in multiple sclerosis patients [37]. For autoimmune diseases, the median TCR β-chain frequency in RA patients was increased tenfold, indicating marked contraction of the repertoire, and disease activity of RA was negatively correlated with the TCR repertoire diversity of CD4+ T cells [4, 38]. Even for infection disease, TCR repertoire was explored the function profile of T cells in HIV patients and the characteristics of immune landscape after injection of HIV vaccine [6, 39]. TCRβ-CDR3 were correlated with disease prognosis of hepatitis B and C [5, 40, 41]. DBS-based methodology provides a simple and quick approach to access changes of immunity and treatment effect in different disease areas.

Each individual has more than 10^9^ T cells which express unique heterodimeric T-cell receptors as identified by the CDR3 peptide sequence of the TCRβ chain. This high diversity provides defense against foreign pathogens. Hou et al. reported that on average two individuals can share 4.85 ± 2.5% of their DNA sequences and 12.17 ± 0.81% of their CDR3 amino acid sequences [42]. According to the current study, less than 1% of uCDR3 clones are shared between two different people except when a parent–child relationship exists (Table 2). Putintseva et al. reported that the degree of overlap was always slightly higher for related individuals for all CDR3s, but this difference never approached a significant level compared to unrelated individuals [43]. In a parent–child relationship, 50% of their HLA alleles are shared. Exposure to the same pathogens may result in specific T cell expansion. Because V-gene usage is highly influenced by human leukocyte antigen (HLA)-type, certain V-genes may be preferentially selected by the immune system, ultimately allowing more shared CDR3s among closely related individuals. The high individual specificity detected by this method allows researchers to look for public CDR3s in a homogenous group or identify disease signatures, based on shared unique CDR3 peptides.

**Fig. 7:**
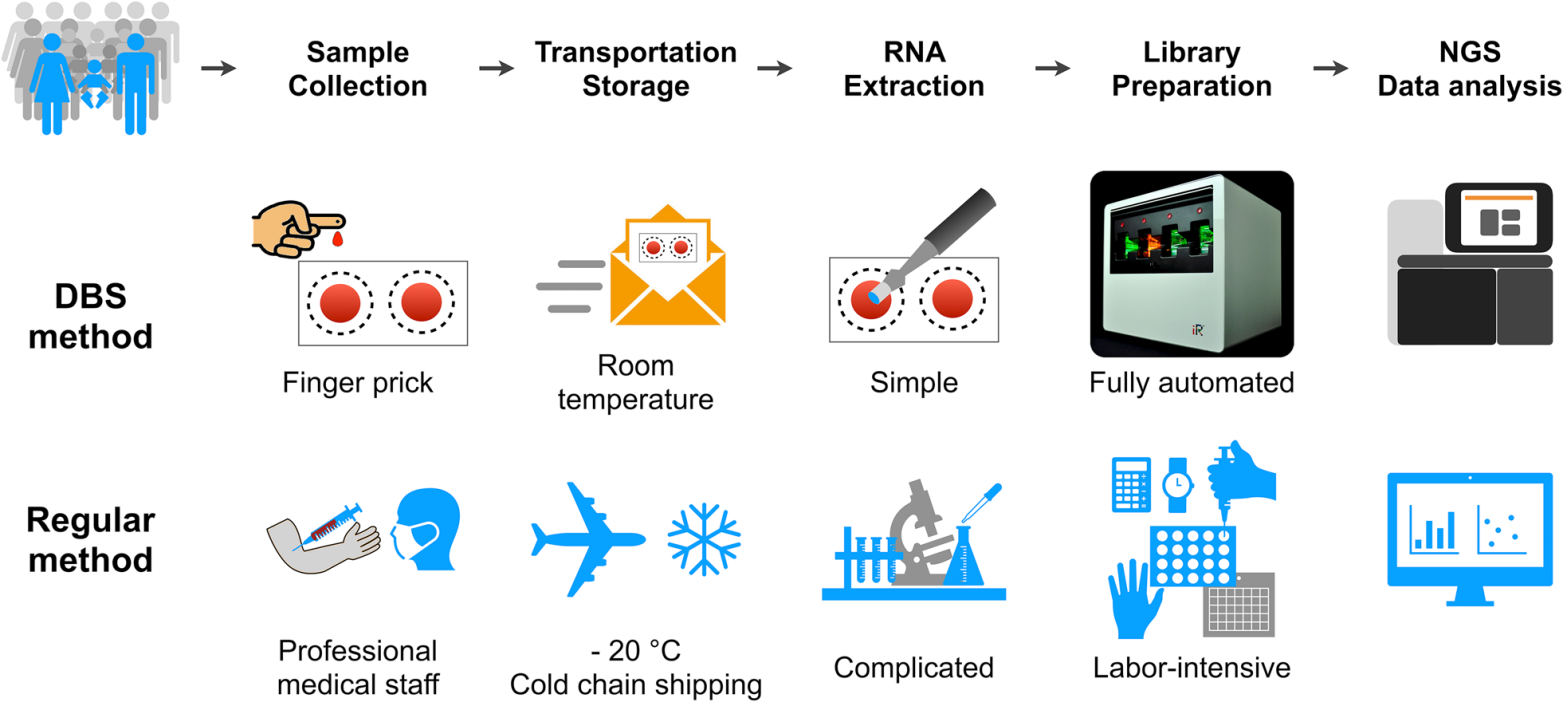
Schematics for the comparison of the methods for TCR repertoire discovery by using a DBS sample and peripheral blood sample. DBS provides a minimally invasive sample collection and convenient alternative to venipuncture. High quality RNA is extracted from DBS-based samples for future molecular amplification and analysis. The entire process of multiplex PCR amplification and library preparation of the immune repertoire is automated in a fully closed cassette, which allows for a less labor-intensive manual process, improves result consistency and prevents contamination amplicon within the laboratory environment

Figure 7 demonstrates the advantages of the DBS-based method for TCR repertoire analysis. DBS provides a minimally invasive sample collection alternative to venipuncture. It is convenient for storage and transportation, in contrast to the necessary of refrigeration of − 20 °C for some RNA-compatible vacutainer tubes, and cold chain shipping for whole blood. Then, high quality RNA can be extracted from DBS-based samples for future molecular amplification and analysis. The entire process of RT-PCR amplification and library preparation of the immune repertoire was conducted automatically in a fully closed cassette, which is easier than the labor-intensive manual process. It also helps improve result consistency and to prevent contamination of the laboratory environment with amplicon. Although other products (PAXgene Blood RNA Tube (IVD) (Qiagen), Tempus™ Blood RNA Tube, etc.) can be used for storage and transportation of RNA at room temperature, refrigeration is still necessary for long-term storage. In addition, they are more expensive and laborious than DBS-based processing due to the requirement for a phlebotomist and specialized extraction RNA kits.

## Conclusions

The current DBS-based method shows high correlation to dominant TCRβ CDR3 clones detected from whole blood. The minimally invasive, cost effective, and convenient storage conditions will allow for the scale-up of surveillance studies or treatment response. More importantly, the development of such a tool allowed collecting of real-world data, i.e., when symptoms showed up, rather than doctors available for an appointment.

## Additional file

**Additional file 1: Table S1.** The statistical results of sample (blood volume (µL)) calculation; **Table S2.** The numbers of shared CDR3 among the different people.

## Abbreviations

NGS: next generation sequencing
TCR: T-cell receptor
5′RACE: 5′Rapid amplification of cDNA ends
DBS: dry blood spots
RA: rheumatoid arthritis
HIV: human immunodeficiency virus
uCDR3: unique CDR3s
β-ME: β-mercaptoethanol
IMGT: ImMunoGeneTics
DI: Diversity Index
*R*^2^: the coefficient of determination
2D: two-dimensional
SPSS: Statistical Package for the Social Sciences
ANOVA: one-way analysis of variance
*EGFR*: epidermal growth factor receptor
*KRAS*: Kirsten rat sarcoma 2 viral oncogene homolog
WHO: The World Health Organization
MHC: major histocompatibility complex
