# Supplementary material for: High throughput sequencing of T-cell receptor repertoire using dry blood spots
Source: J Transl Med. 2019 Feb 18;17:47. doi: 10.1186/s12967-019-1796-4 (PMC6379990; doi:10.1186/s12967-019-1796-4)
Supplement: Supplementary file 1 — Additional file 1: Table S1. The statistical results of sample (blood volume (µL)) calculation; Table S2. The numbers of shared CDR3 among the different people. [file 12967_2019_1796_MOESM1_ESM.docx]

**Additional Table S1 The statistical results of sample (blood volume (µl)) calculation**

| **Dominant  60%** |  |  |  |  |  |  |
| --- | --- | --- | --- | --- | --- | --- |
| **Confidence Interval**  **Confidence Level** | **5%** | **6%** | **7%** | **8%** | **9%** | **10%** |
| **90%** | 254 | 178 | 131 | 101 | 80 | 65 |
| **95%** | 356 | 250 | 185 | 143 | 113 | 95 |
|  |  |  |  |  |  |  |
| **Dominant  70%** |  |  |  |  |  |  |
| **Confidence Interval**  **Confidence Level** | **5%** | **6%** | **7%** | **8%** | **9%** | **10%** |
| **90%** | 223 | 156 | 115 | 59 | 70 | 57 |
| **95%** | 313 | 220 | 162 | 125 | 99 | 81 |
|  |  |  |  |  |  |  |
| **Dominant  80%** |  |  |  |  |  |  |
| **Confidence Interval**  **Confidence Level** | **5%** | **6%** | **7%** | **8%** | **9%** | **10%** |
| **90%** | 171 | 119 | 88 | 68 | 54 | 44 |
| **95%** | 240 | 168 | 124 | 96 | 76 | 62 |

**Additional Table S2** The numbers of shared CDR3 among the different people.

|  |  | P1 | P2 | P3 | P4 | P5 | P6 | P7 |
| --- | --- | --- | --- | --- | --- | --- | --- | --- |
|  | **Unique CDR3** | 1534 | 1547 | 3990 | 3479 | 3826 | 8104 | 2091 |
| P1 | 1534 |  | 1 | 2 | 3 | 1 | 3 | 2 |
| P2 | 1547 | 1 |  | 2 | 2 | 3 | 12 | 0 |
| P3 | 3990 | 2 | 2 |  | 20 | 5 | 8 | 10 |
| P4 | 3479 | 3 | 2 | 20 |  | 2 | 13 | 4 |
| P5 | 3826 | 1 | 3 | 5 | 2 |  | 16 | 3 |
| P6 | 8104 | 3 | 12 | 8 | 13 | 16 |  | 10 |
| P7 | 2091 | 2 | 0 | 10 | 4 | 3 | 10 |  |
